# An Insight into the Abiotic Stress Responses of Cultivated Beets (*Beta vulgaris* L.)

**DOI:** 10.3390/plants11010012

**Published:** 2021-12-23

**Authors:** Seher Yolcu, Hemasundar Alavilli, Pushpalatha Ganesh, Muhammad Asif, Manu Kumar, Kihwan Song

**Affiliations:** 1 Faculty of Engineering and Natural Sciences, Sabanci University, Istanbul 34956, Turkey; muhammad.asif@sabanciuniv.edu; 2 Department of Bioresources Engineering, Sejong University, Seoul 05006, Korea; 3 Department of Plant Biotechnology, M. S. Swaminathan School of Agriculture, Centurion University of Technology and Management, Odisha 761211, India; pushpabhagyalakshmi@gmail.com; 4 Department of Life Science, College of Life Science and Biotechnology, Dongguk University, Seoul 10326, Korea; manukumar007@gmail.com

**Keywords:** beet cultivation, abiotic stress, alkaline, cold, heat, heavy metals, stress tolerance, ultraviolet radiation

## Abstract

Cultivated beets (sugar beets, fodder beets, leaf beets, and garden beets) belonging to the species *Beta vulgaris* L. are important sources for many products such as sugar, bioethanol, animal feed, human nutrition, pulp residue, pectin extract, and molasses. *Beta maritima* L. (sea beet or wild beet) is a halophytic wild ancestor of all cultivated beets. With a requirement of less water and having shorter growth period than sugarcane, cultivated beets are preferentially spreading from temperate regions to subtropical countries. The beet cultivars display tolerance to several abiotic stresses such as salt, drought, cold, heat, and heavy metals. However, many environmental factors adversely influence growth, yield, and quality of beets. Hence, selection of stress-tolerant beet varieties and knowledge on the response mechanisms of beet cultivars to different abiotic stress factors are most required. The present review discusses morpho-physiological, biochemical, and molecular responses of cultivated beets (*B. vulgaris* L.) to different abiotic stresses including alkaline, cold, heat, heavy metals, and UV radiation. Additionally, we describe the beet genes reported for their involvement in response to these stress conditions.

## 1. Introduction

Economically important cultivated beets such as fodder beets, sugar beets, garden beets (e.g., red beet), and leaf beets (e.g., Swiss chard) belong to the sub-species *Beta vulgaris* L. ssp. *vulgaris* [1,2]. All beets originate from a halophytic plant, *Beta vulgaris* L. ssp. *maritima* (sea beet or wild beet), also known as *Beta maritima* L. [3]. Among them, leaf beets and garden beets are used as vegetables [2,4], fodder beets as animal feed [1,2], and sugar beets serve as the source of sucrose, bioethanol, biodegradable polymers, and biofertilizers [5,6,7,8]. In addition to these advantages, beets such as Swiss chard and red beet are a rich source of pigments, termed betalains [9,10,11,12]. Cultivation of beets is widely distributed throughout Turkey and Mediterranean and European countries [13]. Fodder beet plants, which grow at a temperature between 8 °C and 25 °C [1], are cultivated in coastal areas of many countries [14] as well as continental habitats [15]. Wild beet (*Beta maritima* L.) is especially distributed along the coasts of Mediterranean Sea and the European North Atlantic Ocean [3], and it shows significantly higher salt tolerance during germination and seedling stages when compared to other beet varieties [15,16,17,18,19]. Although previous reports have shown genetic diversity in beet species, due to insufficient genetic variation in cultivated beets [15,20,21], the use of wild beet can provide a remarkable source of genetic variability for crop improvement under stressful conditions [20].

Crop plants are subjected to various abiotic stresses, resulting in loss of yield or decreased productivity. Plants have different adaptive and protective strategies at morphological, physiological and molecular levels to cope with environmental stress conditions [21]. Although stress conditions negatively affect beet growth, yield, and quality, the beet cultivars are able to tolerate abiotic stress conditions such as salinity, drought, cold, heat, and heavy metals [18,22,23,24,25,26,27,28]. Sugar beets exhibit tolerance to cadmium (Cd) and are capable of accumulating heavy metals such as Cd and nickel (Ni) [27]. The improvement of beet varieties with better heat tolerance is also an important task due to climate change and global warming [29]. Therefore, we need breeding techniques and agronomic practices for better tolerance to biotic and abiotic stresses in beets [30]. Thus, cultivated beets and their wild ancestor are important genetic sources for crop breeding programs and studying abiotic stress tolerance [15,31]. In the present review, we summarize the morpho-physiological, biochemical, and molecular alterations in cultivated beets (*B. vulgaris* L.) under alkaline, cold, heat, heavy metal, and UV stresses.

## 2. Responses of Cultivated Beets (*B. vulgaris* L.) to Different Abiotic Stresses Including Alkaline, Temperature, Heavy Metal, and UV

Although several studies report different responses of beet cultivars to environmental stresses, research articles and reviews mostly focus on salt and drought response mechanisms in beets [22,23,32,33,34]. However, a comprehensive review describing the responses of cultivated beets to several abiotic stress factors including cold, heat, alkaline, heavy metal, and UV is lacking. Therefore, this review focuses on the responses of cultivated beets (*B. vulgaris* L.) to alkaline, cold, heat, heavy metal, and UV stresses at morpho-physiological, biochemical, and molecular levels. In Table 1, we demonstrate the list of beet genes known for their involvement in response to alkaline, cold, and heavy metal stress.

### 2.1. Alkaline Stress

Alkaline stress (high pH) is one of the abiotic constraints of plants, which co-exists with salt stress and elicits severe detrimental damages to global agricultural production [44]. Over 954 million hectares of land on the globe is affected by salinity [45]. Salt stress results from a neutral salt such as NaCl. Although alkaline salt stress is a type of salt stress, it is caused by alkaline salts such as NaHCO_3_ and Na_2_CO_3_, which is shortly called alkaline stress and causes more damage than neutral salt [46,47]. Numerous research groups across the globe have been perusing tolerance mechanisms to understand the salt stress responses in various crops and model land plants [48,49]. However, the studies focused on high salinity together with alkaline stress are minuscule [44,46]. Apparently, when the plants simultaneously encounter high salinity and high pH, their cumulative damage is more severe than their single occurrence [44]. Several previous reports determined that sugar beet can sustain moderate exposure to saline and alkaline conditions [35,37,48]. However, only a few reports investigated the responses of beets under alkaline stress conditions [47,50]. Hence, to alleviate the alkaline stress-induced damages in commercially important crops such as beets, we need to build a comprehensive knowledge repository that helps devise better strategies for generating stress-tolerant cultivars to attain sustainable agriculture [35,51,52]. Furthermore, developing high salinity-resistant cultivars will efficiently and rationally utilize salinity-affected areas in cultivated lands [45].

Although alkaline stress and salt stress share many common features, such as osmotic stress and ion toxicity, the alkaline condition has unique differences to consider as a different stress form [51]. The alkaline stress includes three principle factors that negatively impact plant growth and development: high soil pH, Na^+^ toxicity, and water deficiency [51]. For example, it has been shown that alkaline stress-induced Na^+^ toxicity and oxidative stress decreased photosynthesis and growth in tomato plants. Moreover, alkaline stress led to higher Na^+^/K^+^ ratio and lower K^+^ content in tomato seedlings [51], and the expression of genes encoding Na^+^ transporters such as *SlNHX1*, *SlNHX2*, *SlSOS1*, *SlHKT1,1*, and *SlHKT1,2* were found to increase in tomato roots exposed to NaHCO_3_ [52,53]. However, we still do not know how sugar beet plants maintain Na^+^-K^+^ homeostasis under alkaline stress conditions and whether Na^+^ transporters contribute to the alkaline stress response in beets. High alkaline pH causes the occurrence of oxidative stress through reactive oxygen species (ROS) and the production of malondialdehyde (MDA), which damage the membrane integrity and intracellular components in plants [47]. To decrease the ROS-induced oxidative stress, plants use several enzymatic and non-enzymatic antioxidants [54]. Enzymatic antioxidants including superoxide dismutase (SOD), catalase (CAT), peroxidase (POX) and ascorbate peroxidase (APX) are involved in scavenging of superoxide radicals and hydrogen peroxide (H_2_O_2_) [54,55,56]. Under salt stress, cultivated beets and wild beet show higher antioxidant enzyme activities [57,58,59]. Similarly, Zou et al. [30] reported that the alkaline stress-tolerant beet cultivar KWS0143 displayed higher antioxidant enzyme activities such as CAT and APX than the sensitive cultivar Beta464 under the same growth conditions [30]. This implies that the tolerant plants are bestowed with durable antioxidant defense equipped with APX, CAT and SOD enzymes to circumvent the cellular damages under salt-alkaline stress [30]. Hence, we need to identify genetic resources with a strong innate antioxidant defense system to fortify beet cultivars with alkaline stress tolerance. In addition to oxidative stress, soils with high pH perturb the macro and micronutrient balance in the soil, which drives the plant to a physiological depression [50]. Previously, Oster et al. [60] classified the alkaline stress into three categories based on the alkaline salt percentage in soil. According to this classification, the alkalinity is considered as mild (3% salt content and pH 7.1–8.5), moderate (3–6% salt and the pH is 8.5–9.5), and severe (>3–6% salt and the pH over 9.5) [60]. In contrast to the detrimental effects of alkaline stress, mild alkaline stress can help the plants to grow bigger and healthier [50,61]. Likewise, in a recent report, Geng et al. [50] examined the differential proteomic responses of sugar beet seedlings by treating them with pH 5, pH 7.5, and pH 9.5 (acidic, neutral, and alkaline) conditions. In the study, they found that the acidic pH caused more growth retardation and enzymatic aberrations than that of neutral and alkaline pH conditions [50]. In contrast to other reports, the alkaline conditions (pH 9.5) significantly improved plant height, fresh weight, total leaf and root area, net photosynthetic rate, stomatal conductance, intercellular CO_2_ concentration, and chlorophyll contents compared to neutral and acidic soils [50]. Moreover, a few more reports found that mild alkaline stress caused better growth, leaf chlorophyll contents, photosynthetic index, and antioxidant activities in sugar beet seedlings [30,61]. Geng et al. [61] found that neutral salt (NaCl:Na_2_SO_4,_ 1:1, Na^+^ 100 mM) remarkably decreased growth and photosynthesis when compared with mild neutral salt (NaCl:Na_2_SO_4_, 1:1, Na^+^ 25 mM) and alkaline conditions (Na_2_CO_3_, Na^+^ 25 mM) in sugar beet plants. In contrast, plants displayed a significant increase in total biomass, leaf area, and photosynthesis under mild neutral salt and alkaline conditions [61]. Interestingly, sugar beet plant growth was not impacted by high alkaline salt (Na_2_CO_3_, Na^+^ 100 mM) as compared to control [61]. We speculate that by virtue of being tolerant to mild saline–alkaline stress, the sugar beet cultivars might display better growth, and we need further experimental evidence to learn the growth patterns of different beet cultivars altered under mild alkalinity. Nevertheless, the growth retardation of plants is found to be proportionately elevating along with the increase in alkaline stress severity [62]. Additionally, alkaline stress responses in plants are usually governed by a multigenic effect, but not by a single gene expression, which implies the intricate stress signaling mechanism [36,63,64].

Numerous reports suggest that under alkaline stress, several physiological parameters, including stomatal conductance (Gs), transpiration rate (Tr), relative water content (RWC), water use efficiency (WUE), accumulation of photosynthetic pigments, and the net photosynthetic rate (Pn), were dropped [47,62]. Specifically, the photosystem-II (PSII) quantum efficiency (Fv/Fm) ratios are negatively affected by alkaline stress, which reduce the electron transport rate [65]. Furthermore, high alkaline conditions dampen the leaf area (LA) and chlorophyll contents (Chl a and b), specifically Chl b, which lowers the photosynthetic rate and WUE [66]. All these physiological parameters will eventually curtail the seedling growth and seedling emergence under alkaline stress [30,62]. In another study, Liu et al. [66] assessed the physiological responses of white Swiss chard under saline and alkaline conditions. Their study identified that although Swiss chard retains higher RWC under alkaline stress, the seedlings suffered from alkaline stress in terms of plant growth. The growth retardation was likely caused by high pH, CO_3_ ^2−^, and HCO_3_^–^ toxicity [66]. Additionally, the physiological indicators such as chlorophyll contents, WUE, and the ionic balance were also perturbed in Swiss chard under 50–100 mM alkaline stress [66]. While comparing the glycine betaine (GB) and proline levels, they found that the GB levels in sugar beet were lower in 50 mM alkaline stress than that of 50 mM salt stress, whereas they did not find any significant alterations in proline levels [66]. This bolsters the notion that the GB plays a more critical role in mediating the alkaline stress tolerance than proline for Swiss chard [66]. It is a well-known fact that compatible solutes including GB, proline, and soluble sugars are remarkably increased under salt stress conditions to maintain photosynthesis and stomatal conductance in beets [67,68,69]. 

In addition to physiological and biochemical responses of beets under alkaline stress, only few genes have been reported to be involved in alkaline stress response in beets. For example, Wu et al. [35] identified 58 putative *WRKY* genes in the sugar beet genome, and among them, nine genes were found to be responsive to the alkaline stress stimulus (~15 mM to 100 mM NaCHO_3_) in both root and shoot tissues [35]. In the study, they found augmented expression of the *BvWRKY10* gene in shoots and *BvWRKY16* expression in root tissues under alkaline stress [35]. The differential expression of *BvWRKY* genes in different tissues implies their functional roles in mediating the alkaline stress responses in different tissues and needs further experimental attention. The WRKY family of transcription factors is plant-specific and plays many critical roles in diverse aspects of plant physiological processes, including abiotic stress responses [70]. Through a transcriptomic approach, some of the differentially expressed genes (DEGs) were shown in alkaline stress-treated beets. Recently, Zou et al. [36] identified differential expression of 1270 genes in alkaline stress-tolerant cultivar KWS0143 in response to alkaline stress. They irrigated the plants with 75 mM alkaline solution (Na_2_CO_3_:NaHCO_3_, 1:2, pH 9.67) and harvested the leaf tissues three (short-term) and seven days (long-term) after the treatments [36]. Compared to the control groups, the short-term and long-term treatments induced the expression of ‘*Ethylene-insensitive protein 2*’ (*LOC104884677*) and ‘*Metal tolerance protein 11*’ (*LOC104886952*) genes, respectively [36]. The results suggest that some of these DEGs would be useful for developing alkaline-tolerant beet cultivars. In another report, Zou et al. [47] assessed the roles of long non-coding RNAs (lncRNAs) in sugar beets under different alkaline stress conditions as previously described in Zou et al. [36] by high-throughput RNA sequencing [47]. In this study, they identified 93 differentially expressed alkaline stress-responsive IncRNAs. Furthermore, additional functional attribution of candidate target genes revealed their association with diverse biological processes, including kinase activity, ribosomal and ribonucleoprotein constituents, and protein metabolic activity, and denotes the association of specific target genes with lncRNAs [47]. In addition, Zou et al. [71] treated the sugar beet seedlings with an alkaline solution and performed small RNA sequencing [71]. They found 53 novel microRNAs (miRNAs) responsive to long-term and short-term alkaline stresses [71]. Similarly, the gene ontology (GO) analysis uncovered enrichment of miRNAs related to the “redox process” and they reported the involvement of ‘*polyphenol oxidase*’ (LOC04900758) gene as the target of alkali-responsive miRNAs. In addition to this, the other 29 miRNAs responsive to long-term alkaline stress can be useful as potential targets to fortify crops with alkaline stress resistance. In Table 2, we summarize the alkaline stress responses in sugar beet varieties.

### 2.2. Cold and Heat Stresses

Because plants are sessile organisms, the ambient temperature has a profound impetus on their entire life cycle, reflecting on their spatial distribution and seasonal behaviors [72]. Their surrounding temperatures also influence the plant growth rate and development, and each plant system has its own set of minimum, optimum, and maximum range of temperatures for survival [73]. Crop production varies depending on the severity of temperatures [74]. Furthermore, plants differentially respond to cold or heat stress according to their developmental stage. Hence, to circumvent the yield damages associated with capricious climates, we need to accumulate the morpho-physiological responses for individual crop varieties. Furthermore, more studies should be performed in order to characterize stress-responsive genes and determine the molecular mechanisms under low and high-temperature stresses in beets, as we have limited knowledge on beet responses to temperature changes.

#### 2.2.1. Cold Stress

Low temperature is one of the most important constraints, impeding plant growth, distribution, biological activity, production, and, ultimately, economic yield [75]. The sensitivity and responses of sugar beet to cold temperatures depend on its developmental stage. Cold is known to drive several developmental events in sugar beet in early and later stages, such as germination, growth, bolting, and accumulation of molassigenic products in the roots [76]. In sugar beets, exposure to cold temperatures at the early seedling stages causes severe root growth retardation and reduced sugar yield [75,77]. Although cold temperatures (i.e.,−2 °C) result in loss of cotyledon viability, the seedlings at 3–4 leaf stage can withstand freezing temperatures up to −10 °C [78,79]. Furthermore, sugar beet roots and shoots show differential responses to cold stress. For instance, in three sugar beet genotypes (GT1, GT2, and GT3), cold temperatures impacted taproot growth more than the shoot growth [80]. It has been reported that there are variations in cold stress tolerance and sensitivity among *Beta* germplasms [81]. Hence, to generate cold-tolerant varieties in commercially essential crops such as beets, knowledge pertaining to their responses to cold conditions is the most important prerequisite [82]. In some geographical sections, sugar beet seeds are sown in early autumn to expose them to shallow winter temperatures (below 0 °C). This practice helps protect the sugar beets from pathogen *Cercospora* attacks and drought stress [75]. Such an early seed sowing in fall, also known as “autumn sowing”, was reported to produce sugar beets with better field emergence than the spring-sown beets [76]. Nevertheless, prolonged exposure of sugar beets at the young seedling stage to extreme cold temperatures seriously limits the yield [75]. Cold-treated sugar beet plants displayed a decrease in photosynthetic efficiency, quantum yield of PSII, leaf CO_2_ concentration, CO_2_ assimilation rate, and leaf transpiration rate [40,80]. Moreover, compatible solutes such as glucose, fructose, and raffinose in leaves were increased by 0 °C and 4 °C cold treatments [40,80], but decreased in taproots in response to freezing temperature [40]. Consistently, under freezing conditions, the sucrose content decreased in roots, followed by leakage of the root sap due to cell alteration in membrane permeability and infection with microbes. Water infiltration due to rapid freezing/thawing can also lead to softening of the root tissue and gradual rotting [83]. Rodrigues et al. [80] reported an interesting finding for the first time. Vernalization (long-term cold treatment at 4–15 °C) leads to a reversal of phloem translocation from taproots (sink tissue) to shoots (source tissue). Redirection of sugar flux is required for induction of flowering in sugar beet. This process might be the reason for the sugar beet sensitivity to freezing temperatures [80]. In a very recent work, three sugar beet genotypes (GT1, GT2, and GT3) were evaluated for freezing tolerance. Freezing temperatures caused the production of ROS, raffinose accumulation, and transcription of genes involved in raffinose metabolism in leaves and taproots [40]. These results suggest that raffinose metabolism has a protective role against freezing injury in sugar beet. Moreover, ROS-scavenging enzymes including SOD and CAT significantly enhanced in response to 4 °C [40]. Consistently, the maximum expression levels of genes encoding antioxidant enzymes such as CAT, APX, ascorbate reductase, and glutathione peroxidase (GPX) were seen at 4 °C, but the expression was reduced at 0 °C. The findings indicate the temperature-dependent ROS production in sugar beet plants.

To date, very few sugar beet genes that function in cold stress response have been functionally characterized under cold stress conditions. In some reports, the transcript levels of genes involved in photosynthesis and compatible solute biosynthesis were investigated in cold-treated beets. For example, Rodrigues et al. [80] reported a sharp increase in the expression of photosynthesis-related genes encoding rubisco activase, rubisco small subunit, a chlorophyll a/b binding protein, and plastocyanin under cold stress. Kito et al. [39] isolated and characterized two sugar beet genes, *B. vulgaris RS1* and *RS2* (*BvRS1* and *BvRS2*), encoding raffinose synthase, which is involved in raffinose biosynthesis. The transcript levels of *BvRS1* and *BvRS2* genes were induced by cold stress in sugar beet leaves and roots [39]. Similarly, in a very recent study, the transcript abundances of galactinol synthase encoding genes, *GOLS2* and *GOLS3*, and two *RS* genes, *BvRS2* and *BvRS5*, were increased by freezing temperature [40]. Surprisingly, the expression of *BvRS5* gene and raffinose amounts remarkably induced in the taproots of freezing-tolerant beet cultivars, GT2 and GT3, but not in the sensitive one, GT1. As compared to other beet genotypes, the GT2 showed the maximum expression levels of *GOLS* and *RS* genes and raffinose levels in taproots, indicating the highest freezing tolerance in GT2 [40]. These findings suggest that the survival of taproot tissue under cold stress might depend on the accumulation of raffinose. As compatible solutes and antioxidants, raffinose family oligosaccharides have important roles in plant response to abiotic stress and stabilizing membranes and proteins [84,85]. In addition to genes involved in raffinose metabolism, the *B. vulgaris Integral Membrane Protein (BvIMP)* gene is the closest homolog of *A. thaliana early response to dehydration-like 6 (AtERDL6)*, which was previously reported for its cold stress-responsive function [86]. Cold stress may lead to elevations in the transcription of *BvIMP* gene and vacuolar sugar trafficking in sugar beet leaves, which is critical for cold stress response and seed germination [37]. Ectopic overexpression of *BvIMP* in *Arabidopsis* resulted in altered glucose concentration during cold conditions, lower accumulation of monosaccharides, and cold-sensitive phenotype compared to the wild-type [37]. In a recent study, Porcel et al. [38] uncovered and isolated a novel endoplasmic reticulum-located aquaporin gene, *B. vulgaris COLD1 (BvCOLD1)*, which is specific to the *Chenopodiaceae* subfamily. The *BvCOLD1* gene is ubiquitously expressed in all tissues of sugar beet [38]; however, its expression was not changed by cold stress [38,75]. In contrast to the wild-type plants, overexpression of *BvCOLD1* restored the membrane fluidity in transgenic *Arabidopsis* lines under cold temperatures and rendered tolerance to cold stress, suggesting that it could be a useful gene for developing biotechnological strategies in order to generate cold-tolerant beet cultivars [38]. 

#### 2.2.2. Heat Stress

Elevated temperatures and water deficit conditions tend to elicit similar impacts on plant water content where the evaporation exceeds the water intake, eventually leading to the plant wilting [87]. Across the globe, we face rapid climate changes and adverse weather problems; hence, developing heat-tolerant crops is the need of the hour. High temperatures impede many vital developmental events such as seed germination and impact seed vigor and viability and seedling emergence, and eventually challenge their survival [88,89]. Critical physiological processes, including photosynthesis and PSII activity, were also affected due to electron transport chain block under heat stress [90,91]. Of late, sugar beet cultivation is also expanding to the tropical and sub-tropical areas, and more people pay attention to cultivation of the sugar beets in summer [29,92]. Ironically, there are few studies aimed to select the heat-tolerant sugar beet cultivars. For the identification of the heat-tolerant beet genotypes, currently, there are no universally approved criteria. Different research groups used different parameters to evaluate the heat stress tolerance in different beet cultivars. For instance, Malmir et al. [92] considered the seed vigor index and root length as evaluation parameters of heat stress tolerance in the early growth stage [92]. To investigate the effects of heat on early growth in sugar beet, they compared 31 sugar beet genotypes under heat stress conditions. Among all the variants tested, the tolerant genotype displayed relatively higher germination, seed vigor, plumule length, and seedling length compared to other genotypes, suggesting that the tolerant one is a prospective cultivar to expand the sugar beet cultivation to tropical areas [92]. Under high temperatures, the leaf temperature, which is associated with vapor pressure deficit (VPD) and stomatal conductance, is known to be enhanced [93]. Moreover, another recent study showed the stress tolerance index (STI) and average root and recoverable sugar yields as selection parameters to identify heat-tolerant lines among 18 sugar beet breeding lines [29]. Among them, six lines were found to have the highest yield, and two lines can sustain under heat stress [29]. In a previous work, two fodder beet cultivars (Ecdogelb and Ecdorot) were used to reveal the impacts of different light intensities and temperatures on fodder beet physiology [94]. High temperature affected root weight ratio (RWR), dry leaf weight (DLW), dry root weight (DRW), total dry weight (TDW), specific leaf area (SLA), net assimilation rate (NAR), and relative growth rate (RGR) in both cultivars at low light intensity [94]. For example, under high light intensity and temperature (20 °C), the cultivar Ecdorot exhibited enhancements of leaf weight ratio (LWR). The highest RGR, RWR, and DLW levels were recorded in response to high temperature and low light intensity in both cultivars. High temperatures result in increments of the growth in root crops, but adversely impact the final biomass [95]. When the temperature was increased from 14 °C to 19.6 °C, an increase in the SLA was also observed [94]. Leaf area, which is used as a selection parameter of drought-tolerant beet cultivars, determines the plant growth rate during initial phase of development [96] and is associated with root and sugar yield [32]. Thus, we assume that the leaf area could be an important parameter to enhance sucrose yield of beets under high temperature conditions.

Unfortunately, so far, no beet genes have been functionally characterized under high temperature conditions. Moreover, the knowledge on beet physiological and biochemical responses is very limited. Hence, comprehensive studies should be performed in different beet cultivars under heat conditions to gain a better understanding of heat tolerance mechanisms in beets at different developmental stages. In Table 3, we summarize the low and high temperature stress responses in cultivated beets.

### 2.3. Heavy Metal Stress

Generally, heavy metals are a group of metals and metalloids with atomic density more than 5 g cm^−3^, or five times or more, greater than water [97], including lead (Pb), cadmium (Cd), nickel (Ni), cobalt (Co), iron (Fe), zinc (Zn), chromium (Cr), arsenic (As), silver (Ag), and the platinum group elements. Mining and smelting operations and agriculture have caused heavy metal contamination of soils with Cd, copper (Cu), and Zn in many areas of the world [98]. Moreover, due to vigorous mining and industrial activities, the metal pollution in soils is becoming prevalent day by day and posing a severe threat to ecological balance [99,100]. For example, in 2002, 22,000 t of Cd, 93,900 t of Cu, 783,000 t of Pb, and 1,350,000 t of Zn were released into the environment on the global scale [101,102]. The buildup of heavy metals in arable lands results in contamination of soils, making them unsuitable for cultivation of plants, including beets. Therefore, the need for collecting scientific information regarding effects of various heavy metals on plants, response mechanisms of plants to heavy metal stress, and agronomic management of this stress can not be overemphasized. 

Exposure of plants to toxic levels of heavy metals causes various metabolic and physiological alterations depending on the metal of concern, level of stress, plant species, cultivar, and other biotic and abiotic factors [103,104,105]. Most of the mineral ions such as Zn, Ni, manganese (Mn), etc., are required for all metabolic activities in plants at miniscule amounts. However, if the metal ion presence exceeds the threshold, they tend to exert detrimental effects on plant metabolism, resulting in leaf chlorosis, necrosis, turgor loss, a decrease in the rate of seed germination, and a crippled photosynthetic apparatus, which could cause plant death [106,107,108]. Among the heavy metal ions, Cd, Zn, and Cu are reported as the most toxic metals, with serious health hazards to humans when they infiltrate the food chain [109]. Like other plants, heavy metals adversely affect the sugar beet as they proscribe various metabolic activities [27,110,111]. For example, heavy metals such as Pb damage the vacuolar membrane in red beet taproots [112]. Lead is one of the most toxic metals for plant cells, and it negatively affects plant growth, photosynthesis, respiration, and membrane transport [113]. Cd treatment in *B. vulgaris* caused growth retardation, leaf chlorosis, and increased root/whole plant ratio [114] with decreased root-tip respiration and photosynthesis [110,114]. As compared to control plants, Cd-treated plants exhibited lower shoot dry weights, photosynthetic pigments, and reduction in water content of shoots and fine roots, dramatically [114]. Direct application of Cd on isolated leaves, protoplasts, and chloroplasts inhibited CO_2_ fixation without affecting the PSI or PSII and dark respiration rate, whereas indirect Cd application through the culture medium decreased the maximal quantum yield of CO_2_ assimilation [110]. Papazoglou and Fernando [27] tested the growth and heavy metal tolerance of sugar beet plants in Cd- and Ni-contaminated soil [27]. They found that the highest Ni concentration (20 g) was lethal to the plants, and an interesting fact they found was that the single application of Ni caused higher toxic effects than the combination of Ni and Cd [27]. Nevertheless, the combination of Cd (5 g) and Ni (10 g) treatment resulted in a drastic reduction in fresh and dry biomass of aerial parts and beets, and a decrease in plant height [27]. Very recently, Haque et al. [43] found that toxic levels of Cd cause growth retardation of sugar beet plants because of low iron levels resulting in photosynthetic inefficiency, and cellular oxidative stress [43]. Cd-treated plants displayed sensitivity to oxidative stress, leading to an increase in levels of O_2_^−^ and H_2_O_2_ in roots and shoots. In addition, Haque et al. [43] examined the antioxidant defense system in sugar beet under heavy metal stress and found that Cd stress caused an enhancement of CAT enzyme activity in the shoots, whereas the activities of other antioxidant enzymes such as SOD, APX, and GR did not change in neither roots nor shoots. Furthermore, the results from a previous study indicated reduced uptake of N, P, Mg, K, Mn, Cu, and Zn upon Cd toxicity [114]. Similar to Cd stress, Zn toxicity decreased macronutrient concentrations (N, K, and Mg), whereas it enhanced the P level in shoots as well as roots [115]. In sugar beets, Cu and Zn treatments also significantly reduced plant growth, shoot and root lengths, and dry weight [116]. At high Cu concentrations, the shoots showed turgor loss, but lower Cu concentration did not affect plant growth [116]. Sagardoy et al. [115] reported that the toxic level of Zn reduced water content, leaf numbers, and root/shoot ratio, along with wrinkled and chlorotic leaves in sugar beet [115]. Root proteome analysis of sugar beet showed slight changes in metabolism under low and mild Zn levels, but higher levels of Zn led to cell death and cessation of metabolism through decreasing aerobic respiration and damaging defense systems required for oxidative stress response. Thus, the results showed that toxic Zn levels caused damages to the oxidative stress defense mechanisms due to Zn competition with divalent cations such as Fe, which might strengthen the symptoms of Zn toxicity in plants [117]. In summary, the results denote that the degree of toxicity of heavy metals on plant metabolism depends on plant species, the duration of stress, and type and concentration of heavy metals they were exposed to [111].

Several studies highlighted foliar uptake of heavy metals and their effects on the membrane permeability through the cuticle and percentage of open stomata in sugar beet [118,119]. A previous study demonstrated that sugar beet seedlings grown in nutrient solution containing high concentrations of CdCl_2_ showed an increased leaf transpiration rate and a decreased stomatal aperture area. Thus, higher Cd concentrations affected the permeability of the leaf cuticle [119]. Apart from seedlings, Cd stress was also shown to negatively influence sugar beet taproot growth. For instance, long-term Cd exposure caused decreased sucrose uptake and diminished dry weight in taproots, but the direct addition of Cd^2+^ to the medium enhanced the sucrose uptake at the tonoplast [120]. Increased accumulation of Cd lowered the contents of glucose, fructose, and sucrose in both shoots and roots of sugar beet [121] and inhibited the activity of plasma membrane H^+^-ATPase (PM H^+^-ATPase) [122]. Additionally, in several studies, changes in the activity of enzymes related to metal homeostasis and nitrate metabolism were investigated in heavy metal-treated sugar beets. For instance, the activity of ferric chelate reductase (FCR) involved in iron homeostasis was decreased under short-term exposure of Pb and Cd, but prolonged exposure increased the FCR activity in sugar beet roots [123]. Recently, Haque et al. [43] reported that the reduction in FCR activity and expression of *iron-regulated transporter 1 (BvIRT1)* gene suggested a negative impact of Cd in Fe acquisition. In another study, the Pb-treated sugar beet plants exhibited altered Cu deficiency levels and increased FCR activities [114]. When sugar beet plants were exposed to the highest concentrations of heavy metals (Ni and Cd), the nitrate content and nitrate reductase (NR) activity dramatically dropped in the leaves [111]. 

To cope with heavy metal stress, plants have developed certain strategies involving two type of mechanisms, i.e., avoidance and tolerance [124]. The avoidance mechanisms emphasize on limiting the uptake of heavy metals (e.g., Cd) into the plant, whereas tolerance refers to storing (e.g., in vacuoles) and accumulation of heavy metals by binding it to peptides, amino acids, and proteins [125,126]. To limit uptake of heavy metals and detoxify them, plants have developed certain mechanisms, including the development of morphological structures such as thick cuticle and cell walls, mycorrhizal symbiosis, and biologically active tissues such as trichomes [127,128,129]. Sugar beet, like canola, is a non-mycorrhizal plant species, and therefore has a limited ability to phytostabilize heavy metals and has been suggested as a source of phytoremediation of heavy metals [130,131] despite the negative effects of heavy metals on beet growth, physiology, and metabolism. For instance, among different crop plants tested, red beets have the capacity of removing Cd from soils [130]. It has been reported that sugar beet plants have the ability to accumulate Ni, Pb, and Cd [27,132]. Papazoglou and Fernando [27] suggested that sugar beet could be a suitable crop for phytoextraction of Cd as it can accumulate Cd and produce biomass. Similarly, Yadav et al. [132] compared several crops for their capacity to accumulate heavy metals and found that sugar beets accumulated the highest amount of Cd and Pb among the studied crops. These findings clearly suggest that sugar beet could be an efficient source for phytoremediation of heavy metal-contaminated soils. Since heavy metals such as Cd and Pb have serious effects on human and animal health, sugar beets grown on heavy metal-contaminated soils must not be used for food and feed purpose, but only for industrial purposes such as bioethanol production. Due to the hazardous nature of heavy metals, heavy metal-contaminated areas are of limited use, and removal strategies of excessive heavy metals from soils are required [133]. Phytoremediation is a promising approach to dampen the toxic effects of heavy metal pollution by utilizing the artificial hyperaccumulators. Transgenic plants, which can take up the persistent heavy metals, serve as artificial hyperaccumulators. For instance, Liu et al. [131] found an important role of glutathione (synthesized by *γ*-glutamylcysteine synthetase-glutathione synthetase) in cellular tolerance of heavy metal stress. Overexpression of *γ-glutamylcysteine synthetase-glutathione synthetase (StGCS-GS)* gene from *Streptococcus thermophilus* in sugar beet plants showed the explicit role of *StGCS-GS* in enhancing Cd, Zn, and Cu tolerance and accumulation of these metals in shoots of transgenic sugar beets [131]. Transgenic lines also displayed resistance to different heavy metal combinations, i.e., 50 μM Cd-Zn, Cd-Cu, Zn-Cu, and Cd-Zn-Cu, and had higher levels of glutathione (GSH) and phytochelatin (PC) compared to the WT [131]. Moreover, a study by Dronnet et al. [134] concluded that the sugar beet pulp is economical and highly selective in binding of divalent metal cations such as Cd^2+^, Cu^2+^, Ni^2+^, Pb^2+^ and Zn^2+^; thus, it could be useful as a substrate to entrap heavy metals in aqueous solution. Surprisingly, it was reported that the intake of juice extracted from red beet roots protects the chickens from Cd-induced oxidative stress with enhanced immune power [135]. However, it is unfortunate that the response mechanisms of cultivated beets and wild beet to heavy metal stress is yet to be investigated in detail. Further comprehensive studies are necessary to examine the influences of heavy metal contamination on different beet cultivars, and yield and quality of bioethanol [27]. In addition, only few genes have been reported for their involvement in heavy metal response in beets. For instance, two MTP genes, *BmMTP10* and *BmMTP11* encoding metal-tolerant proteins from wild beet (*B.* *maritima*), were found to render tolerance to high concentrations of Mn^2+^ when expressed in yeast cells. Transcript level of *BmMTP10* gene was augmented by the presence of excessive Mn^2+^, but *BmMTP11* transcription was not altered, suggesting that BmMTP10 and BmMTP11 proteins have non-redundant functions in Mn detoxification [41]. Thus, the study demonstrated that the BmMTP10 protein, which is localized to the Golgi apparatus, is specific to Mn^2+^ transport and decreased Mn^2+^ levels in yeast cells [42]. Ni detoxification was regulated by a couple of genes in *B. maritima* named as toxic nickel concentration (NIC), i.e., *NIC3*, *NIC6*, and *NIC8* [42]. It was speculated that all three genes are involved in tolerance to Ni toxicity. Yeast cells expressing a cDNA clone (NIC6) from *B. maritima* showed substantially high tolerance to Ni but not to the other heavy metals such as Co, Cd, and Zn [42]. Even though the excess Ni accumulation is toxic to plants, *B. maritima* plants overcome the Ni-induced toxicity by internal sequestration, but not by effluxing Ni [42]. In a very recent study, under Cd stress, sugar beet roots displayed higher levels of putative *inactive Cd/Zn-transporting ATPase (BvHMA3)* and *natural resistance-associated macrophage protein 3* (*BvNRAMP3*) gene expression, suggesting that these genes might participate in Cd uptake [43]. Interestingly, in response to Cd application, no significant changes have been observed in the expression of *phytochelatin 3 (BvPC3)* gene encoding PCs [43], which are involved in the detoxification of Cd [136].

Further studies on sugar beet are needed to investigate the physiological, cellular, and molecular alterations induced by heavy metals to help plant biologists develop breeding strategies to improve sugar beet cultivars with efficient phytoremediation ability and ability to grow in heavy metal stress-affected fields [43]. In Table 4, we summarize the heavy metal stress responses in beets.

### 2.4. Ultraviolet (UV) Stress

Ultraviolet radiation (UV) causes various changes in metabolic activities of plants, imposing malfunctions and retarded overall growth. The key processes in plants affected by UV radiation include photosynthesis, biomass, respiration, transpiration, etc. UV-B (280–320 nm) radiation becomes a serious threat to the organisms because of the reduction in stratospheric ozone [137]. The stress triggers changes at molecular level by protein degradation, altering the double helical structure of DNA and antioxidant contents, etc. However, under UV stress conditions, plants adopt defensive tolerant mechanisms [138,139].

We have very limited information about the physiological and biochemical responses of beets to UV stress. Moreover, there are no reports on the molecular mechanisms and genes involved in UV stress response in beets. A report by Panagopoulos et al. [140] demonstrated that the leaves of sugar beets curled inwards and positioned towards light source with 68% growth reduction over control (ROC) under yellow light, whereas the plants were dead under a combination of yellow light and UV-B after three weeks [140]. They found that some parameters such as leaf area, fresh and dry weights, and total chlorophyll levels in sugar beet were decreased under UV radiation [140]. On the other hand, carotenoid concentrations showed different patterns upon imposition of UV radiation. For example, yellow light and a combination of white light + UV-B resulted in higher carotenoid contents, suggesting the protective role of these pigments against photo-oxidation [140]. The study also showed an increase in leaf peroxidase activity under the combination of white light and UV-B [140]. The increased peroxidase activity and ultraweak luminescence upon UV-B exposure and ascorbic acid incubated leaves represents a strong correlation in *Hibiscus* leaves [141] and sugar beet [140]. In a recent study, the most widely cultivated Iranian sugar beet variety, BR1, was used to analyze biochemical and physiological responses against different doses (3.042, 6.084, and 9.126 kJm^−2^d ^−1^) of UV-B radiation [142]. The UV-B-treated sugar beet plants showed a drastic growth retardation with reduction in fresh weight, dry weight, and height. Moreover, total chlorophyll and carotenoid contents and photochemical efficiency of PSII were reduced in UV-treated plants. Interestingly, no significant raise in the proline levels was noticed. Betalain levels increased by 8%, 28%, and 34% with increased UV-B radiation of 3.042, 6.084, and 9.126 kJm^−2^d ^−1^, respectively, indicating that these water-soluble pigments possess tolerant metabolic function in sugar beet varieties against UV-B radiation. Hence, it is likely that the BR1 variety is a suitable plant material for areas with UV-B irradiation [142]. 

Levall and Bornman [143] showed the establishment of a reproducible regeneration technique in sugar beet, wherein production of somaclonal variations was observed and UV-B-tolerant plants were selected. After additional UV-B treatment, unselected somaclones displayed significantly higher UV damage and lower carotenoid levels than the selected plants [143]. The UV irradiation exposure in in vitro conditions exhibited more tolerant callus parts than the protoplasts, paving the way for the selection of UV-tolerant sugar beet somaclones [143]. In another study, Levall and Bornman [137] showed differences between *Cercospora*-sensitive and -tolerant sugar beet plants upon the combined biotic (*Cercospora* fungal infection) and abiotic (UV radiation) stresses. The line tolerant to fungal infection was shown to be tolerant to UV-B alone and combined UV-B and biotic stresses; however, the photosynthetic yield significantly reduced in the sensitive line [137]. A report by Bornman et al. [144] showed that the UV-B radiation was not capable of penetrating organelles such as chloroplasts, resulting in intact thylakoids [144]. On the other hand, the ultrastructural image of sugar beet leaves showed prominent damages due to UV-B radiation (290–320 nm), whereas UV-C (254 nm)-treated sugar beet plants showed fewer structural changes, leading to a higher quantity of starch in chloroplasts, grana stacks fused to each other, and decreased damage to the leaf surface [145].

The results described above suggest that beet plants are adversely affected by UV stress conditions at the morpho-physiological level. However, molecular mechanisms and UV stress-responsive genes in beets are still elusive. Further studies are needed to better understand the UV stress response mechanisms at the morpho-physiological, biochemical, and molecular levels in different beet cultivars. In Table 5, we summarize the responses of cultivated beets to UV radiation.

## 3. Concluding Remarks

As an economically important crop plant, cultivated beets have multifarious industrial applications ranging from food and nutrition to sugar and bioethanol production. Despite beet tolerance to different abiotic stresses [16,24], the cultivation of beets is often challenged by various adverse environmental factors [34]. These climatic abnormalities are anticipated to be more aggravated due to human industrial activities as well as global warming effects. Hence, to meet the global food security demands, developing stress-resilient plant genotypes is one of the most important topics for crop production in stress-affected fields. However, selection of the suitable beet genotypes tolerant to environmental conditions is an arduous task for plant breeders [29] as there is no clear and comprehensive understanding about the stress signaling pathways and tolerance mechanisms in different climatic regions. Even though our understanding of the heavy metal accumulation ability of beets is limited, sugar beet plants have been suggested as a candidate for phytoremediation [28,126,135]. Sugar beets grown in contaminated soils pose a serious threat to human and animal health. Therefore, use of sugar beets grown for phytoremediation must be limited to industrial purposes, such as bioethanol production. Furthermore, we have limited experimental data showing the molecular mechanisms underlying the stress response of *B. vulgaris* genotypes under extreme temperatures (cold and heat), UV radiation, high pH, and heavy metals. Although the beet cultivars show some degree of stress resistance, persistent exposure to these abiotic constraints takes a toll of their development and growth potential. On the other hand, the wild beet (*B. maritima*) displays better stress tolerance compared to the modern beet cultivars as it is rich in allelic diversity [18,34]. Most likely, the modern cultivars lost some of their stress tolerance traits during progressive domestication. While utilizing the genetic variability in wild beet and stress-tolerant beets, we can ameliorate the allelic diversity, which further eases the improvement of tolerant varieties. 

**Figure 1:**
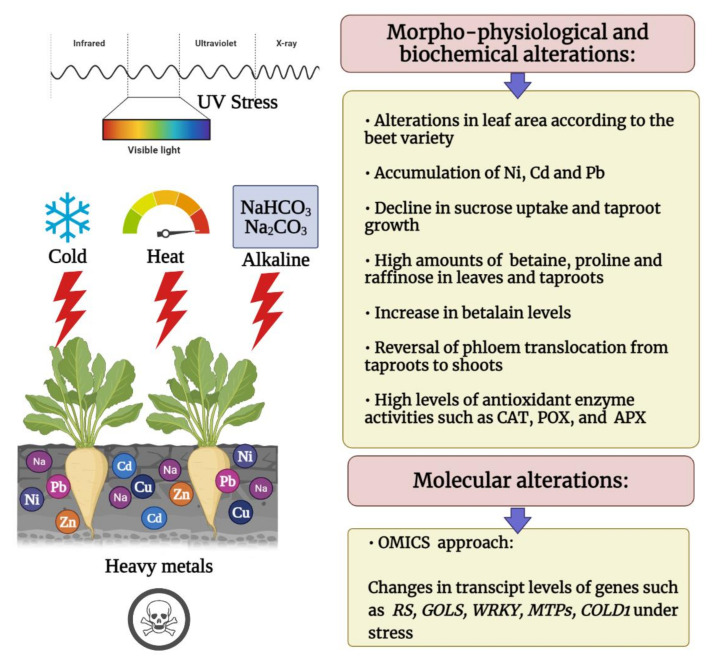
Schematic representation of morpho-physiological, biochemical, and molecular alterations in beets under alkaline, cold, heat, heavy metal, and UV conditions. This figure was created via BioRender.com (accessed on 12 December 2021).

Since several beet cultivars were introduced and acclimated to tropical and sub-tropical climates, it would be thus essential to establish the pan-genomic studies of beet cultivars to uncover the precise genetic modifications responsible for the ecological adaptations. Establishing the phenotypic and genotypic diversity of various beet cultivars grown in different climatic zones by utilizing the modern bioinformatic advents can enable us to generate stress-resistant crops. Consequently, further investigations are necessary to design breeding strategies under abiotic stress, and compare stress response mechanisms and signaling pathways between cultivated beets and wild beet. In Figure 1, we summarize morpho-physiological, biochemical, and molecular changes in beets under different abiotic stresses including alkaline, cold, heat, heavy metals, and UV radiation.

## Figures and Tables

**Table 1:** Beet genes known to be involved in response to alkaline, cold, and heavy metal stresses.

Type of Abiotic Stress	Gene Name	References
Alkaline stress	WRKY transcription factor family (*WRKY10* and *16*)	[35]
Alkaline stress	Metal Tolerance Protein 11 *(MTP11)*	[36]
Alkaline stress	Ethylene-insensitive protein 2 *(EIN2)*	[36]
Alkaline stress	Polyphenol Oxidase *(PPO)*	[36]
Cold stress	Integral membrane protein *(IMP)*	[37]
Cold stress	A novel ER-located aquaporin gene *(COLD1)*	[38]
Cold stress	Raffinose synthase 1 and 2 (*RS1* and *RS2*)	[39]
Freezing	Galactinol synthase 2 and 3 (*GOLS2* and *GOLS3*) Raffinose synthase 2 and 5 (*RS2* and *RS5*)	[40]
Heavy metal	Metal tolerance protein (*BmMTP10* and *BmMTP11*)	[41]
Heavy metal	Toxic nickel concentration *(NIC3*, *NIC6* and *NIC8)*	[42]
Heavy metal	Natural resistance-associated macrophage protein 3 *(NRAMP3)*	[43]

**Table 2:** Alkaline stress responses in cultivated beets.

Beet Variety	Stress Treatments	Experimental Results	Reference
*B. vulgaris*, KWS0143	NaHCO_3_:Na_2_CO_3_ (0.5%, 0.7%, 0.9%)	High activity levels of antioxidant enzymes, such as CAT and APX	[30]
*B. vulgaris*, H004	pH 5, pH 7.5, and pH 9.5	Acidic pH resulted in more growth retardation, photosynthesis, and enzymatic aberrations than neutral and alkaline pH	[50]
*B. vulgaris*, KWS0143	75 mM alkaline solution (NaHCO_3_:Na_2_CO_3_, 2:1, pH 9.67)	Significant inhibition of plant growth	[47]
		A decrease in stomatal conductance (Gs), transpiration rate (Tr), and net photosynthetic rate (Pn)	
		Identification of 93 differentially expressed alkaline stress-responsive IncRNAs	
*B. vulgaris*, H004	Neutral salt (NaCl:Na_2_SO_4,_ 1:1) and alkaline salt (Na_2_CO_3_)	Mild neutral salt and alkaline conditions led to a significant increase in total biomass, leaf area, and photosynthesis	[61]
*B. vulgaris*, KWS0143 and Beta464	0, 25, 50, 75 and 100 mM of mixed (Na_2_CO_3_:NaHCO_3_, 1:2) alkaline conditions	The levels of photosynthetic pigments were remarkably diminished by high alkaline stress (75 and 100 mM)	[62]
		Sugar beet displayed resistance to alkaline stress through osmotic adjustment and antioxidant enzymes under mild alkaline stress	
*B. vulgaris* L. var. *cicla*	50 and 100 mM alkaline salt (NaHCO_3_ and Na_2_CO_3_, 9:1)	Growth retardation due to high pH, CO_3_ ^2−^, and HCO_3_^–^ toxicity	[66]
		Lower GB levels under 50 mM alkaline stress than 50 mM salt stress, whereas no significant alterations in proline levels	
*B. vulgaris*, Gantang7	0, 15, 25, 50 and 100 mM NaHCO_3_	Among 58 putative *WRKY* genes, 9 genes were found to be responsive to alkaline stress (~15 mM–100 mM NaCHO_3_) in both root and shoot	[35]
		Enhanced expression of *BvWRKY10* gene in shoots and *BvWRKY16* expression in roots under alkaline conditions	
*B. vulgaris*, KWS0143	75 mM alkaline solution (Na_2_CO_3_:NaHCO_3_, 1:2, pH 9.67)	Differential expression of 1270 genes in alkaline stress-tolerant cultivar KWS0143 under alkaline stress	[36]
*B. vulgaris*, KWS0143	75 mM alkaline solution (Na_2_CO_3_:NaHCO_3_, 1:2, pH 9.67) for short-term (3 d), and long-term (7 d)	53 novel miRNAs responsive to long-term and short-term alkaline stress	[71]

**Table 3:** Cold and heat stress responses in cultivated beets.

Beet Variety	Stress Treatments	Experimental Results	Reference
*B. vulgaris*, Merak, and Antic cultivars	Cold stress (0 °C, 5 °C and 10 °C)	Some parameters, such as proline content, F_v_/F_m_ ratio, and root dry matter, were higher in cold-tolerant varieties than sensitive ones	[77]
		Genetic diversity in cold tolerance of sugar beet cultivars was observed at seedling stage	
*B. vulgaris*, Bianca	Cold stress (−2 °C)	Prolonged exposure of sugar beets at the young seedling stage to the cold stress seriously limits the yield	[75]
		After short-term cold stress, transcription factors and genes involved in metabolic pathways were expressed in sugar beet leaves and roots	
*B. vulgaris*	Cold stress (−2 °C and −10 °C)	Sugar beet plantlets at the cotyledon stage completely died at −2 °C; however, at the 3–4 leaf stages, the plants can survive up to −10 °C	[78,79]
*B. vulgaris*	Cold stress (−5 °C)	Freezing injury results in an increase in tonoplast permeability for sucrose	[83]
		Under freezing conditions, the sucrose content decreased in roots, followed by leakage of the root sap due to cell alteration in membrane permeability and infection with microbes	
*B. vulgaris*, NK-210 mm-0	Cold stress (4 °C)	The transcript levels of two sugar beet genes, *B. vulgaris RS1* and *RS2* (*BvRS1* and *BvRS2*), encoding raffinose synthase, were induced by cold stress in sugar beet leaves and roots	[39]
*B. vulgaris * genotypes; GT1, GT2, and GT3	Cold stress (12 °C, 4 °C, and 0 °C)	Raffinose accumulation and transcription of genes involved in raffinose metabolism in leaves and taproots have been observed under low temperature	[40]
*B. vulgaris*, belladonna	Cold stress (4 °C)	Ectopic overexpression of *BvIMP* in *Arabidopsis* led to altered glucose concentration under cold conditions, lower accumulation of monosaccharides	[37]
*B. vulgaris*	Cold stress (10 °C)	Overexpression of *BvCOLD1* restored the membrane fluidity in transgenic *Arabidopsis* lines under cold stress and rendered tolerance to cold	[38]
*B. vulgaris* var. *altissima* Döll	Heat stress (20 °C and 30 °C)	Among 31 sugar beet genotypes, the tolerant genotype exhibited higher germination, seed vigor, plumule length, and seedling length under heat stress	[92]
*B. vulgaris*, USKPS25 and USC944-6-68 breeding lines	High temperature conditions in the field experiments	The stress tolerance index (STI) showed positive correlation with average root and sugar yields, which were used as selection parameters to identify heat-tolerant lines	[29]
*B. vulgaris* var. crassa Mansf. Fodder beet cv. Ecdogelb and Ecdorot	Heat and cold stress (18.28, 19.58, 18.26, 17.61, and 14.1 °C)	Two fodder beet cultivars showed the highest levels of RGR, RWR, and DLW under high temperature and low light intensity	[94]

**Table 4:** Heavy metal stress responses in beets.

Beet Variety	Stress Treatments	Experimental Results	Reference
*B. vulgaris*, red beet	0.1–100 μM trimethyllead chloride (Met_3_PbCl)	Lead (Pb) damage the vacuolar membrane in red beet taproots	[112]
*B. vulgaris*, Monohill	10 μM and 50 μM Cd-EDTA or CdCl_2_	As compared to control plants, Cd-treated plants showed lower shoot dry weights, photosynthetic pigments, and reduction in water content of shoots and fine roots	[114]
		The reduction in uptake of N, P, Mg, K, Mn, Cu, and Zn due to Cd stress	
*B. vulgaris*, Monohill	Direct Cd application (1, 5, 20, 50, 2000 μM CdCl_2_) Indirect Cd application (5, 10, 20 μM CdCl_2_)	Direct application of Cd on isolated leaves, protoplasts and chloroplasts inhibited CO_2_ fixation, whereas indirect Cd application through the culture medium decreased the maximal quantum yield of CO_2_ assimilation	[110]
*B. vulgaris*	0; 0.5; 5; 10 g Cd 0 1; 10; 20 g Ni Cd + Ni (0 + 0, 0.25 + 0.5, 2.5 + 5, 5 + 10)	The highest Ni concentration (20 g) is lethal to the plants	[27]
		The single application of Ni causes higher toxic effects than the combination of Ni and Cd	
*B. vulgaris *	10 μM CdSO_4_	Cd stress causes growth retardation in sugar beets because of low iron levels resulting in photosynthetic inefficiency, and oxidative damage	[43]
		Sugar beet roots displayed higher levels of *BvHMA3* and *BvNRAMP3* gene expression, whereas the reduction in ferric chelate reductase (FCR) activity and expression of *iron-regulated transporter 1 (BvIRT1)* gene was observed	
*B. vulgaris*, Orbis	50, 100, and 300 μM ZnSO_4_	Zn toxicity decreased macronutrient concentrations (N, K, and Mg), whereas it enhanced the P level in shoots as well as roots	[115]
		The toxic level of Zn reduced water content, leaf numbers, and root/shoot ratio along with wrinkled and chlorotic leaves	
*B. vulgaris*, Orbis	50, 100, and 300 μM ZnSO_4_	High levels of Zn led to cell death and cessation of metabolism through decreasing aerobic respiration and damaging defense systems required for oxidative stress response	[117]
*B. vulgaris*, Qaweterna	0.1, 1, 10, 100 μM CuSO_4_, or ZnSO_4_	Cu and Zn treatments significantly reduced plant growth, shoot and root lengths, and dry weight	[116]
		At high Cu concentrations, the shoots showed turgor loss, but lower Cu concentration did not affect plant growth	
*B. vulgaris*, Monohill	0 to 10 μM CdCl_2_	Sugar beet seedlings grown in nutrient solution containing high concentrations of CdCl_2_ showed an increased leaf transpiration rate and a decreased stomatal aperture area. Thus, higher Cd concentrations affected the permeability of the leaf cuticle.	[119]
*B. vulgaris*, Monohill	0, 1, 5 or 20 μM Cd^2+^	Long-term Cd exposure caused decreased sucrose uptake and diminished dry weight in taproots, but direct addition of Cd^2+^ to the medium enhanced the sucrose uptake at the tonoplast	[120]
*B. vulgaris*, Monohill	0, 5 or 50 μM Cd^2+^	Increased accumulation of Cd lowered the contents of glucose, fructose, and sucrose in both shoots and roots	[121]
*B. vulgaris*, Monohill	Short-term application: 10 and 50 μM CdCl_2_/Cd-EDTA, or 1 and 2 mM Pb-EDTA for 30 min and 1 h Long-term application: 10 and 50 μM CdCl_2_, /Cd-EDTA, or PbCl_2_, and 10; 50; 500; 1000 and 2000 μM Pb-EDTA for 7–10 days	The activity of FCR involved in iron homeostasis was decreased under short-term exposure of Pb and Cd, but a prolonged exposure increased the FCR activity in roots	[123]
*B. vulgaris*, hybrid NS Hy-11	10^−4^, 10^−2^, 1 mM NiSO_4_, or CdCl_2_	When sugar beet was exposed to the highest concentrations of heavy metals (Ni and Cd), the nitrate content and nitrate reductase (NR) activity dramatically dropped in the leaves	[111]
*B. vulgaris*, US-8916	0, 50, 100, 200 μM CdCl_2_, ZnCl_2_, or CuCl_2_	Overexpression of *StGCS-GS* from *S. thermophilus* in sugar beets showed the explicit role of this gene in enhancing Cd, Zn, and Cu tolerance and accumulation of these metals in transgenic sugar beets	[131]
*B. maritima*	75 μM NiCl_2_	Yeast cells expressing a cDNA clone (NIC6) from *B. maritima* showed high tolerance to Ni	[42]
		*B. maritima* plants overcome the Ni-induced toxicity by internal sequestration, but not by effluxing Ni	
*B. maritima*, TR 51196	8 mM Mn^2+^ for yeast cells 2 mM Mn^2+^ for gene expression analyses	Two *MTP* genes, *B.* *maritima MTP10* and *MTP11* encoding metal-tolerant proteins, were found to render tolerance to high concentrations of Mn^2+^ in yeast cells	[41]
		Transcript level of *BmMTP10* gene was augmented by the excessive Mn^2+^, but *BmMTP11* transcription was not altered	

**Table 5:** UV stress responses in cultivated beets.

Beet Variety	Stress Treatments	Experimental Results	Reference
*B. vulgaris*, inbred genotype no. 22	Yellow light (350–450 nm) Yellow light + UV-B (350–450 nm + 280–320 nm)	The leaves curled inwards and positioned towards light source with a 68% growth reduction over control under yellow light, whereas the plants were dead under the combination of yellow light and UV-B	[140]
		Yellow light and a combination of white light and UV-B led to higher carotenoid levels	
*B. vulgaris*, BR1	3.042, 6.084 and 9.126 kJm^−2^d ^−1^ of UV-B	The UV-B-treated sugar beets showed a drastic growth retardation with reduction in fresh weight, dry weight, and height	[142]
		Total chlorophyll and carotenoid contents and photochemical efficiency of PSII were reduced, but the betalain levels were increased under UV-B	
*B. vulgaris*, inbred lines S (CCA 242) and T (GGO 480)	13 kJ m^−2^ d^−1^ of UV-B *Cercospora beticola*	The sugar beet line tolerant to *Cercospora* fungal infection was shown to be tolerant to UV-B alone and combined UV-B and biotic stresses, but the photosynthetic yield significantly reduced in sensitive line	[137]
*B. vulgaris*, Primahill, derivative 9164	UV-B (290–320 nm) UV-C (254 nm)	The ultrastructural image of sugar beet leaves showed prominent damages due to UV-B (290–320 nm), whereas UV-C (254 nm)-treated plants showed fewer structural changes, leading to a higher quantity of starch in chloroplasts, grana stacks fused to each other, and decreased damage to the leaf surface	[144,145]
